# Gene variants and educational attainment in cannabis use: mediating role of DNA methylation

**DOI:** 10.1038/s41398-017-0087-1

**Published:** 2018-01-22

**Authors:** Maria Carla Gerra, Subramaniam Jayanthi, Matteo Manfredini, Donna Walther, Jennifer Schroeder, Karran A. Phillips, Jean Lud Cadet, Claudia Donnini

**Affiliations:** 1 Department of Chemistry, Life Science and Environmental Sustainability, University of Parma, Parma, Italy; 2 Molecular Neuropsychiatry Research Branch, NIDA Intramural Research Program, Baltimore, MD USA; 3 Office of the Clinical Director, NIDA Intramural Research Program, Baltimore, MD USA

## Abstract

Genetic and sociodemographic risk factors potentially associated with cannabis use (CU) were investigated in 40 cannabis users and 96 control subjects. DNA methylation analyses were also performed to explore the possibility of epigenetic changes related to CU. We conducted a candidate gene association study that included variants involved in the dopaminergic (*ANKK1*, *NCAM1* genes) and endocannabinoid (*CNR1*, *CNR2* gene) pathways. Sociodemographic data included gender, marital status, level of education, and body mass index. We used MeDIP-qPCR to test whether variations in DNA methylation might be associated with CU. We found a significant association between SNP rs1049353 of *CNR1* gene (*p* = 0.01) and CU. Differences were also observed related to rs2501431 of *CNR2* gene (*p* = 0.058). A higher education level appears to decrease the risk of CU. Interestingly, females were less likely to use cannabis than males. There was a significantly higher level of DNA methylation in cannabis users compared to controls in two of the genes tested: hypermethylation at exon 8 of *DRD2* gene (*p* = 0.034) and at the CpG-rich region in the *NCAM1* gene (*p* = 0.0004). Both genetic variants and educational attainment were also related to CU. The higher rate of DNA methylation, evidenced among cannabis users, may be either a marker of CU or a consequence of long-term exposure to cannabis. The identified genetic variants and the differentially methylated regions may represent biomarkers and/or potential targets for designs of pharmacological therapeutic agents. Our observations also suggest that educational programs may be useful strategies for CU prevention.

## Introduction

Marijuana is the most common drug used illicitly throughout the world. Approximately 9% of those exposed to cannabis become addicted (cannabis use disorder, DSM-5). The number increases to 16% when cannabis use (CU) is initiated during adolescence and to 50% when cannabis is used daily^1^. Because of the gateway hypothesis of early CU^2^ and because of potential negative effects of the drug^3^, genetic risk factors for CU have been investigated using a diversity of methods. Twin studies have suggested that genetic markers may explain about 50% of the variance in CU disorders^4–7^. Association studies have also provided evidence of genetic risks that include differential changes at Taq1A allele (rs1800497, ANKK1) that influences the function of dopamine receptors^8^. In addition, changes at rs1049353, rs806380, rs2180619, rs806379, rs12720071, rs2023239 and the haplotype rs6454674-rs806380-rs806377-rs1049353 in the cannabinoid receptor 1 gene (*CNR1*) and at rs2501431 in the cannabinoid receptor 2 gene (*CNR2*) were found to be associated with working memory dysfunction, enhanced impulsivity, neurocognitive impairments, anxiety disorder, and depression in cannabis users^9–13^.

Interestingly, genome-wide association studies of cannabis users and dependent individuals have also identified several genetic variants that are known to play a role in neurogenesis and dopaminergic neurotransmission. These include* ANKFN1*, *RP11-206 M11.7*, *SLC35G1*, *CSMD1*, *NCAM1*, *CADM2*, *SCOC* and *KCNT2*^14–16^. Whole genome sequencing approaches have also documented variations involved in the regulation of *MEF2B*and *PCCB* genes expression^17^. Moreover, a gene cluster located on chromosome 17q24 [c17orf58, BPTF, PPM1D] was previously reported to be associated with cannabis use disorders (CUD) diagnosed by DSM-5 ^18^.

Several investigators have sought to identify environmental factors and clarify how their interactions with inherited familial risk conditions and related gene variants might impact susceptibility to cannabis abuse. Traumatic and negative life events, including poor parental care, reduced family bonding, childhood maltreatment, physical and sexual abuse, exposure to community violence, low level of school engagement, as well as school drop-outs appear to play important roles in the initiation and persistence of CU^19–24^. Nevertheless, the mechanisms of interactions between these factors and their relationships to CU remain to be clarified.

The picture is rendered more complicated by epigenetic factors that might also trigger the initiation and/or maintenance of CUD^25–30^. Specifically, modifications in DNA methylation have been reported in cannabis users, in tetrahydrocannabinol (THC)-dependent subjects, and in a rat model of prenatal THC exposure to CB1 and CB2^25,29^. These included DNA modifications in catechol-*O*-methyltransferase (*COMT*) gene^26^ and in PACSIN1, a kinase involved in neuron morphogenesis and neurodevelopmental processes^30^. Interestingly, a significant relationship between DNA sequence variants and DNA methylation has also been established^26,31,32^.

Despite the reported association between genetic risks and CU, more remains to be done to further clarify the role that these genetic changes might play in diverse populations. Therefore, the major aim of our study was to identify risk factors that can trigger or exacerbate the clinical course of CU. Towards that end, we sought to investigate the potential role of gene variants affecting dopamine and cannabinoid receptors function, SNP Taq1A (*ANKK1*) and the SNP rs1049353 (*CNR1*) in CU. Second, we also studied interactions of these variants with sociodemographic measures including body mass index (BMI), gender, marital status, and levels of education. Third, we sought to discover if changes in DNA methylation might also play a role in CU.

## Materials and methods

### Subjects

A total of 136 subjects (Table 1), 40 cannabis users (30 males and 10 females) and 96 control subjects (38 males and 58 females), aged 18–60 years, were selected among samples collected and stored previously at the Intramural Research Program (IRP) of the National Institute of Drug Abuse (NIDA), project #12-DA-N472, NIDA, IRP (Health Outcomes by Neighborhood (HON)—Baltimore). These subjects were recruited among people who live in Baltimore City or one of the surrounding areas. Based on frequency of use, the cannabis participants were moderate to heavy users^33^, with no other drug use or alcohol abuse (total *n* = 40; moderate, *n* = 20 and heavy, *n* = 20). Ninety-six (96) unrelated healthy individuals who live in the same geographic area and who have never smoked cannabis served as control participants. The study was approved by the NIDA Addiction IRB. Written informed consent was obtained from all participants. The subjects were reimbursed for their time.

**Table 1:** Socio-demographic data of samples collected (96 CTRLs subjects and 40 MJ users)

		Controls (*n* = 96)		MJ users (*n* = 40)	
		*n*	%	*n*	%
Gender					
Male		38	39.58%	30	75.00%
Female		58	60.42%	10	25.00%
Marital status					
Not married		84	87.50%	38	95.00%
Married		12	12.50%	2	5.00%
Level of education					
1 = some high school/GED		12	12.50%	12	30.00%
2 = H.S. diploma		24	25.00%	10	25.00%
3 = some college		33	34.38%	16	40.00%
4 = college graduate / Masters / Ph.D.		27	28.13%	2	5.00%
BMI					
<25		36	39.56%	18	48.65%
≥25		55	60.44%	19	51.35%
Ethnicity					
African American		63	66.32%	34	87.18%
Asian		2	2.11%	0	0.00%
European American		24	25.26%	3	7.69%
More than one race		5	5.26%	2	5.13%
Native Hawaiian or other Pacific		1	1.05%	0	0.00%
Missing		1		1	

Exclusion criteria included inability to sign informed consent and age <18 years old. Participants in the present study were excluded for other illicit drugs or alcohol. In addition to self-report, the use or abuse of other illicit substances was ruled out by obtaining observed urines that were negative for methamphetamine, 3,4-methylenedioxymethamphetamine, benzodiazepines, cocaine metabolites, methadone, oxycodone, phencyclidine, buprenorphine, and morphine. The participants were screened for prevalence of anxiety disorder (measured by Brief Anxiety Scale (BAS)) and PTSD disorder (measured by PTSD Checklist-Civilian Version (PCL-C)). Only four participants were affected by possible co-occurring anxiety and PTSD disorder. The number was too low to allow another statistical analysis.

### Design

**Fig. 1:**
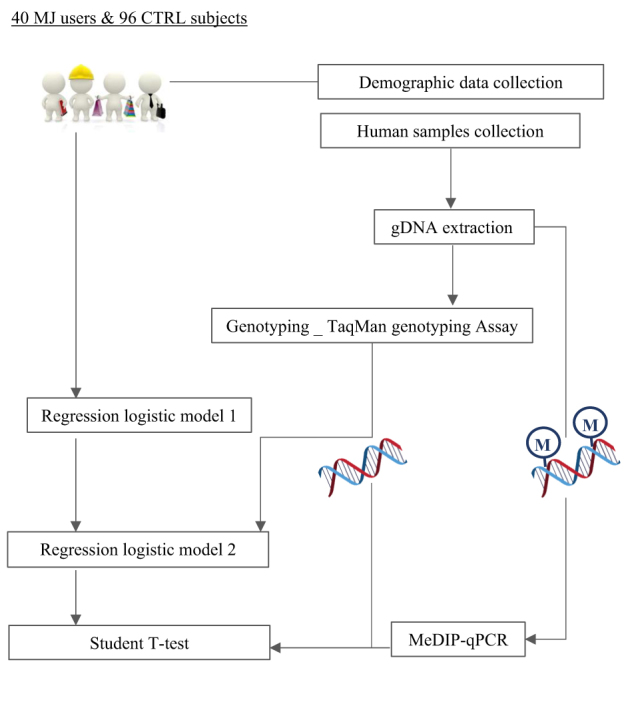
Workflow diagram

The study follows the workflow showed in Fig. 1.

### Sociodemographic measures

Sociodemographic data were collected for each subject (see Table 1). A semi-structured questionnaire was utilized to collect socio-demographic data concerning gender, marital status, educational attainment, BMI, and ethnicity.

### Sample collection and DNA extraction

Peripheral whole blood was previously collected from participants via venipuncture and 5 mL, maintained at −80 °C, were used in the present study (see Supplementary material for preparation of blood samples). DNA extraction was carried out using the QIAamp DNA Blood Midi/Maxi Kit (Spin Protocol, Qiagen). Aliquots of the genomic DNA extracted were used for both genotyping and DNA methylation analysis.

### Genotyping

The polymorphisms related to the genes listed in Table 2A were genotyped in cannabis users and controls. Ten nanograms of genomic DNA were processed with TaqMan Genotyping Assays (Thermo Fisher) for the identification of allelic variants (see Supplementary material for detailed procedure).

**Table 2A:** List of candidate genes and analyzed polymorphisms

Gene	SNP	DNA sequence variation	Position	Functional consequence	Global MAF
*ANKK1*	rs1800497	C/T (REV)	11:113400106	missense: Glu ⇒ Lys	A = 0.3257/1631
*CNR1*	rs1049353	A/G (REV)	6:88143916	synonymous codon: Thr ⇒ Thr	T = 0.1294/648
*CNR1*	rs2180619	A/G (FWD)	6:88168233	upstream variant 2KB	G = 0.4685/2346
*CNR1*	rs806379	A/T (FWD)	6:88151548	intron variant, upstream variant 2KB	T = 0.3952/1979
*CNR1*	rs6454674	G/T (FWD)	6:88163211	intron variant	G = 0.3141/1573
*CNR1*	rs12720071	A/G (REV)	6:88141462	UTR variant 3’	C = 0.0899/450
*CNR1*	rs2023239	C/T (FWD)	6:88150763	intron variant, upstream variant 2KB	C = 0.1779/891
*CNR2*	rs2501431	A/G (FWD)	1:23875153	synonymous codon: Gly ⇒ Gly	G = 0.3466/1736

### DNA methylation analysis

**Fig. 2:**
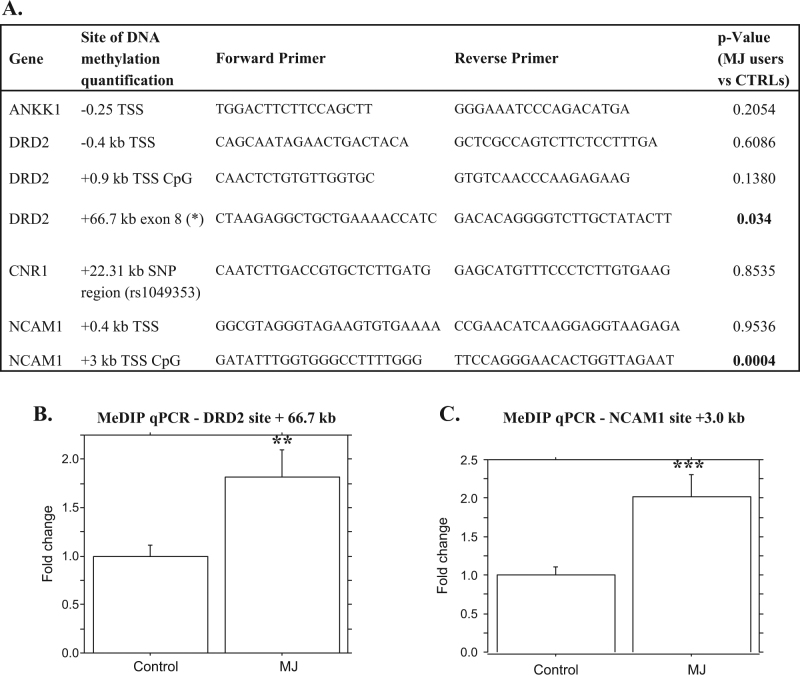
**a** List of the regions where DNA methylation level has been quantified, with related *p*-value, from the comparison between MJ users and CTRLs. (*) This site was designed as close as possible to the Taq1A SNPs, *ANKK1* gene. **b c** Bar plots related to unpaired *t*-test, comparing MJ users and control subjects, for **b** +66.7 kb from TSS, *DRD2* gene and **c** +3 kb from TSS (UCSC Genome Browser on Human Dec. 2013 (GRCh38/hg38) Assembly)

DNA methylation status was analyzed in seven sites, listed in Fig. 2a. These were in regions of *ANNK1*, *CNR1*, and in two genes that belong to the same cluster of *ANKK1* on chromosome 11, *DRD2* and *NCAM1* genes. The primer sequences were designed to include sites as close as possible to the polymorphic SNP Taq1A and to the SNP rs1049353 or to the transcription start site (TSS) including CpG regions and intron−exon junctions.

To quantify DNA methylation in specific sites of genes (listed in Fig. 2a) involved in the neurobiology of CUD, MeDIP-qPCR was performed. The procedure includes three steps^34^: (1) sonication of gDNA (20 µg); (2) immunoprecipitation of the sonicated and denatured gDNA (5 µg); this step was performed overnight at 4 °C using 5 μl of a monoclonal antibody against 5 mC (anti-5-methylcytosine, 5-mC, mouse monoclonal antibody (catalog #33D3, Millipore), 50 µl IP Buffer (10 mm sodium phosphate (pH 7.0), 140 mm NaCl, 0.05% Triton X-100), in a final volume of 500 µl TE; incubated the mixture with 80 µl of Dynabeads (Life Technologies) overnight at 4 °C and washed it three times with 700 µl of IP buffer. The beads were then treated with proteinase K for 3 h at 50 °C and recovered the methylated DNA by phenol-chloroform extraction followed by ethanol precipitation. Sheared “input” DNA samples were collected prior to immunoprecipitation for subsequent comparison with immunoprecipitated DNA (3) quantitative real-time PCR using specific ChIP primers to determine the DNA methylation enrichment. Primers were designed using BLAST Pick up Primer Tool; the CpG regions position was investigated using UCSC genome browser (on Human Dec. 2013 (GRCh38/hg38) Assembly; https://genome.ucsc.edu/cgi-bin/hgGateway). The specific primer sequences used in this study are listed in Fig. 2a and all the details on primer sites annealing are reported in the Supplementary material section.

### Statistical analyses

Fisher’s exact test was applied to examine the relationship between both allele frequencies and genotypic distribution with CU.

Haplotype frequencies, haplotype odds ratio and 95% confidence interval, and pairwise linkage disequilibrium were estimated. Haplotype frequencies were determined by using PLINK (1.07, Author: Shaun Purcell, URL: http://pngu.mgh.harvard.edu/purcell/plink/). The SNPs involved in haplotypes analysis (Table 2C) were the six SNPs of *CNR1* on chromosome 6 (rs1049353|rs806379|rs6454674|rs2023239|rs12720071|rs2180619).

**Table 2C:** Haplotype analysis of the six SNPs of CNR1. Positive associations at the 2–4 SNPs levels are reported

Sliding window	SNPS forming the haplotype	Haplo-type	Frequency in MJ	Frequency in CTRLs	Test for association CHISQ	DF	*p*-value
2 SNPs	rs12720071|rs1049353	TT	0.009868	0.08497	5.279	1	0.02159
	rs1049353|rs2023239	TT	0.00692	0.09218	6.402	1	0.0114
3 SNPs	rs12720071|rs104935| rs2023239	TTT	0.004799	0.0684	4.721	1	0.02979
	rs1049353|rs2023239| rs806379	TTA	0.008201	0.09034	6.019	1	0.01415
4 SNPs	rs12720071|rs1049353|rs2023239|rs806379	TTTA	0.006228	0.07111	4.705	1	0.03007
	rs1049353|rs2023239| rs806379|rs6454674	TTAT	0.009029	0.07984	4.907	1	0.02674
	rs1049353|rs2023239| rs806379|rs6454674	CCTT	0.3776	0.2373	5.339	1	0.02086

Logistic regression models were used as main statistical tool to evaluate the role of potential explanatory factors (gender, marital status (married vs unmarried), educational attainment (1. Some high school/GED, 2. High school diploma, 3. Some college, 4. College Graduate, Masters, Ph.D.)), BMI, on the risk of CU. Two nested models were estimated. The first model is aimed to assess the potential influence of socio-demographic variables on CU, while a second model also included genotyping data. Because of the different gender composition of the two groups, it is considered appropriate to insert gender in the logistic regression models to evaluate its potential confounding effect.

MeDIP-qPCR statistical analysis was performed using STATVIEW 5.0. All the quantitative data are presented as mean + SEM. For data comparing controls (CTRL) and cannabis users (MJ) groups, unpaired Student's *t*-test was used (StatView version 5.01, St. Louis, Missouri).

In addition, to identify a possible interaction between DNA methylation and respectively the SNP rs1049353 and the SNP rs1800497 (the only two variants for which it was possible to design MeDIP primers). Student's *t*-test for independent samples was run.

For all the statistical analyses, results were considered statistically significant at *p* ≤ 0.05.

## Results

### Socio-demographic findings

In the first logistic regression model (Table 3A), evaluating the influence of socio-demographic factors on CU, gender was found to significantly contribute to CU risk. Male participants had 6.6 times higher risk to be cannabis users compared to females (*p* = 0.001). In addition, all the levels of educational attainment higher than the reference category seem to decrease the risk of CU. The highest level of education, College Graduate/Masters/Ph.D., reduced significantly the risk by 90% (*p* = 0.006). Both marital status and BMI did not show any statistical relation with CU.

**Table 3A:** Variable(s) entered on step 1: gender, marital status (married (ref cat)-unmarried), BMI, education (4 categories)

Variables in the equation						
	B	S.E.	Wald	df	Sig.	Exp(B)
Gender, female (ref cat), male	1.889	0.505	14.006	1	**0.000**	6.615
Marital status, married (ref cat), unmarried	1.168	0.919	1.615	1	0.204	3.216
BMI	0.024	0.031	.610	1	0.435	1.024
1-Some high school/2-GED (ref cat)						
3-H.S. diploma (1)	−1.088	0.618	3.095	1	0.079	0.337
4-Some college (2)	−0.354	0.581	.370	1	0.543	0.702
5-College-graduate/6-Masters/7-Ph.D (3)	−2.436	0.883	7.621	1	**0.006**	0.087
Constant	−3.001	1.488	4.068	1	0.044	0.050

Significant contributions are indicated as bold values

### Genetic findings

G allele and homozygous GG genotype of SNP rs1049353 (G1539A) (*CNR1* gene) were significantly higher among cannabis users compared to control subjects (respectively *p* = 0.002; *p* = 0.01) (Table 2B). Even excluding homozygous A/A subjects, due to the low number of observations (0% among cannabis users and 4.2% among controls), the differences were confirmed (*p* = 0.02). Differences were observed (*p* = 0.058) for the SNP rs2501431of the *CNR2* gene. There were no statistical significant differences in the SNP Taq1A (*ANKK1*) or *CNR1* gene (rs806379, rs6454674, rs2023239, rs12720071, rs2180619) (Table 2B).

**Table 2B:** Genotype and allele frequencies

SNP ID (gene)	Genotypes and alleles	Subjects CTRLs	MJ users	Fisher’s exact test
rs1800497 (*ANKK1*)	CC	50,00%	51.28%	0.76
	TT	9.38%	12.82%	
	CT	40.63%	35.90%	
	C allele	87.10%	76.63%	0.88
	T allele	12.90%	23.37%	
rs1049353 (*CNR1*)	GG	78.13%	97.44%	0.01
	AA	4.17%	0.00%	
	GA	17.71%	2.56%	
	G	86.98%	98.72%	0.002
	A	13.02%	1.28%	
rs2180619 (*CNR1*)	AA	21.74%	23.68%	0.9
	GG	34.78%	36.84%	
	AG	43.48%	39.47%	
	A allele	43.48%	43.42%	1
	G allele	56.52%	56.58%	
rs806379 (*CNR1*)	AA	19.79%	17.95%	0.62
	TT	25.00%	33.33%	
	AT	55.21%	48.72%	
	A allele	47.40%	42.31%	0.5
	T allele	52.60%	57.69%	
rs6454674 (*CNR1*)	GG	12.77%	7.50%	0.21
	TT	36.17%	52.50%	
	GT	51.06%	40.00%	
	G allele	38.30%	27.50%	0.09
	T allele	61.70%	72.50%	
rs12720071 (*CNR1*)	CC	21.88%	20.51%	1
	TT	0.00%	0.00%	
	TC	78.13%	79.49%	
	C allele	60.94%	60.26%	1
	T allele	39.06%	39.74%	
rs2023239 (*CNR1*)	CC	10.42%	12.50%	0.47
	TT	48.96%	37.50%	
	TC	40.63%	50.00%	
	C allele	30.73%	37.50%	0.32
	T allele	69.27%	62.50%	
rs2501431 (*CNR2*)	AA	47.37%	58.97%	0.058
	GG	11.58%	0.00%	
	AG	41.05%	41.03%	
	A allele	32.11%	20.51%	0.07
	G allele	67.89%	79.49%	

The haplotype analysis for the six *CNR1* SNPs located on chromosome 6 (rs1049353|rs806379|rs6454674|rs2023239|rs12720071|rs2180619) revealed positive associations for the 2–4 SNPs levels reported in Table 2C.

### Gene-sociodemographic interactions

A second logistic regression model (Table 3B) tested simultaneously the influence of genetic and environmental risk factors considering the concurrent influence on CU of the previous significant parameters: the genetic variables rs1049353 (*CNR1*) and rs2501431 (*CNR2*), gender and educational attainment. Gender (*p* < 0.05), and the educational attainment (*p* = 0.002) were confirmed to have a significant relation with CU. G allele of rs1049353 (*CNR1*) was confirmed to confer a higher risk to be cannabis users (*p* = 0.05).

**Table 3B:** Variable(s) entered on step 2: gender, education (4 categories) rs1049353, A allele (ref cat)-G allele, rs25GA43A, G allele (ref cat)-A allele

Variables in the equation						
	B	S.E.	Wald	df	Sig.	Exp(B)
Gender, female (ref cat), male	2.054	0.514	15.951	1	**0**.**000**	7.797
1-Some high school/2-GED (ref cat)						
3-H.S. diploma (1)	−1.374	0.709	3.757	1	0.053	0.253
4-Some college (2)	−.542	0.623	.757	1	0.384	0.582
5-College graduate/6-Masters/7-Ph.D (3)	−2.851	0.926	9.491	1	**0.002**	0.058
rs1049353, A allele (ref cat) G allele	3.161	1.116	8.024	1	0.005	23.584
rs25GA43A, G allele (ref cat) A allele	−.183	0.503	.133	1	0.716	0.833
Constant	−3.854	1.179	10.683	1	0.001	0.021

Significant contributions are indicated as bold values

### Epigenetic findings

**Fig. 3:**
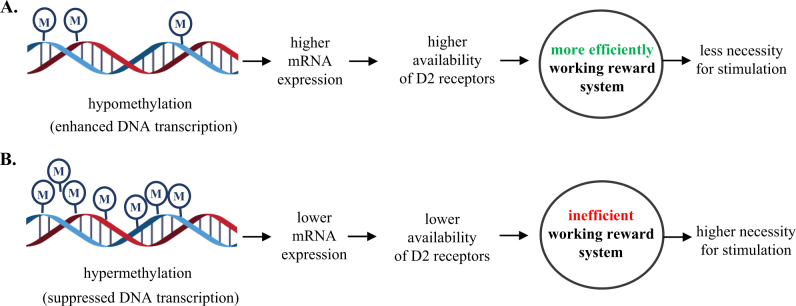
**a** Hillemacher’s (2015) hypothesis on altered DNA-methylation in pathological gambling patients. **b** Based on the current results, hypothesis of DNA hypermethylation in patients with cannabis use disorders

We analyzed DNA methylation status in seven sites of *ANNK1*, *CNR1*, *DRD2* and *NCAM1* genes, listed in Fig. 2a and related to CU. DNA methylation was significantly higher in cannabis users compared to control subjects in two of the regions analyzed (Fig. 2b, c). The first site was in the exon 8 of *DRD2* gene at +66.7 kb from the TSS (see Figure 4S) (*p* = 0.034) and the second was located on a CpG region at +3 kb from the TSS (Figure 6S) in the *NCAM1* gene (*p* = 0.0004). No differences in DNA methylation were found at *ANKK1* −0.25 kb, *DRD2* −0.4 kb and +0.9 kb, *NCAM1* +0.4 kb, *CNR1* +22.31 kb, comparing cannabis users and control subjects (Figs. 1S-3S, 5S, 7S).

## Discussion

The main findings of our study are: (1) SNP rs1049353 in *CNR1* gene is associated with CU; (2) the highest level of education, College graduate/Masters/Ph.D., reduced significantly the risk to be cannabis users; and (3) changes in DNA methylation, comparing cannabis users and control, were observed in two sites related to *DRD2* and *NCAM1* genes.

Our findings are consistent with the nominal association found between the G allele, SNP rs1049353, and cannabis dependence symptoms^10^. The SNP rs1049353 polymorphism appears to play a significant role in the function of cannabinoid receptor by influencing mRNA translation as well as secondary structure and stability of the protein^35,36^. Thus, it is possible that individuals who carry this SNP might experience different marijuana-induced behavioral or physiological effects and may also show different phenotypes that are regulated by the cannabinoid system^10,37–40^. These suggestions might explain why the G allele may serve as a risk factor for CU^41^.

To increase the power to detect genetic traits associated with CU, haplotype-based association analysis was also used. Different combinations of SNPs in *CNR1* showed significant associations with CU at 2–4 SNPs levels for seven haplotypes in our study. These observations are consistent with another research group that had reported a significant association of *CNR1* haplotype rs806368-rs1049353-rs2023239-rs6454674 with the level of cannabis exposure and decreased volume of the right anterior cingulum^42^.

For the variant rs2501431 in the other cannabinoid receptor *CNR2* gene, differences were observed in genotype distribution, with the homozygous A/A genotype being more frequent in the cannabis group. When we excluded GG genotypes, the association did not reach significant values (*p* = 0.7), leading us to assume a very important role of the G allele. This statement is consistent with the report that the GG genotype is associated with alcoholism in a Japanese population^43^. Together, these observations suggest the need for further study to elucidate the role of rs2501431 in *CNR2* gene as possible risk factor of CU and other addiction diagnoses.

In addition, our data highlight the importance of gender in CU susceptibility. Compared to females, male subjects in the study presented a significantly higher risk to use cannabis. These findings underlie the importance of better exploring gender difference in addiction research. Gender differences in CU are indeed frequently reported, with male proneness to use the drug more frequently or at a higher rate than females^44–46^. Gender-based differences in cannabinoid effects have been evidenced by preliminary studies investigating the role of gonadal hormones in the regulation of cannabis receptors density and affinity^47^. Other studies highlighted that sex differences could also be influenced by drug disposition and body fat distribution^48^. However, the fact that females are underrepresented in our study and others may also lead to incomplete and biased results.

Importantly, the evaluation of sociodemographic risk factors helped to confirm that higher education attainment might serve as a possible protective factor, considering the significant inverse relation between education levels and CU. A previous study had reported that users with higher education do not suffer from cognitive deficits with chronic abuse of cannabis^49^. Interestingly, strong engagement in school and good school achievements have also been repeatedly reported to be important resilience factors against substance use disorders^50–54^. Thus, poor school performance and dropping out of schools may not only be risk factors for substance use disorders but may be the consequences of shared genetic/epigenetic risks that predict both school failure and CU initiation^55,56^. The potential impact of cannabis on school performance is also very important to consider^57–60^. Logistic regression analysis demonstrated that both genetic (rs1049353, *CNR1* gene) and sociodemographic risk factors are coincident, representing endophenotypes associated with CU risks. The role of endophenotypes for the rational development of therapeutic and preventive strategies has previously been assessed and suggests that these are important variables in treatment approaches^61^.

Our epigenetic findings also appear to suggest that not only inherited gene variants, but also changes in gene expression could be associated to CU. Hypermethylation was reported in cannabis users compared to controls at *DRD2* exon 8 and in a CpG-rich region of the *NCAM1* gene. The observed NCAM1 differentially methylated region (DMR) was located on the 5′ CpG island of the gene and promoter-associated DMR-CpGs are usually shown to cause transcriptional silencing^62^. Even if the functional significance of DNA methylation in introns, exons, and intergenic intervals is still less well-understood^63,64^, the *DRD2* DMR, located within the exon 8, suggests the importance of these gene body sites, as already shown in a study on parental THC exposure^65^.

Both genes, *DRD2* and *NCAM1* have a central role in the dopaminergic pathway^66^ and have been recently found significantly associated with lifetime CU^16^. They are part of the *NCAM1*–*TTC12*–*ANKK1*–*DRD2* gene cluster (NTAD), suggested to be associated to nicotine dependence^67^ and other substance use disorders in general^68^.

Other behavioral conditions were reported to be associated with DNA methylation in these two genes. *DRD2* promoter showed highest methylation levels in patients with gambling behavior, compared to non-gambling participants and, because DNA-hypermethylation is usually associated with transcriptional repression, the subsequently higher availability of D2 receptor was assumed to result in a more active working of the reward system^69^.

Based on these evidences, we could hypothesize (Fig. 3) that increased DNA methylation observed in cannabis users' blood cells may in some way reflect a lower D2 mRNA expression, lower availability of D2 receptors underlying a reward deficit condition. This difference in the reward system function related to methylation may lead to a reduced need for reinforcing stimuli in non-cannabis users, and a higher necessity of pharmacological stimulation in cannabis-dependent subjects.

The observed hypermethylation in *NCAM1* gene is consistent with the hypermethylated CpG regions of *NCAM1* in alcohol-dependent patients compared with controls^70,71^. This gene that was previously found significantly associated with lifetime CU^16^ encodes a neural cell adhesion molecule implicated in important functions during development and maintenance of the nervous system. A study in rodents proposed NCAM as a modulator of the dopaminergic pathway and a potential pharmacological target for dopamine-related psychiatric disorders^72^.

In the interpretation of our findings concerning hypermethylation in cannabis users, it is impossible to establish any causal relationship. Epigenetic changes affecting *DRD2* and *NCAM1* could be the result of long-term exposure to cannabis and not a pre-existing condition involved in CU neurobiological vulnerability.

The present study has certain limitations. First, because the number of cannabis users was relatively low, statistical power was not as strong as expected. Moreover, since polymorphisms may vary in frequency among different ethnic groups^73^, it might have been more appropriate to split the samples based on ethnic groups. However, this was not possible due to the relatively small number of subjects per group. The results obtained by the present study could thus be attributed to the ethno-racial background of the participants. Thus, any extension to other ethnic groups should be done with caution and will need to be investigated further.

Another important limitation is that most of the changes in DNA methylation patterns were observed in peripheral cells^74^ such as WBCs that may not reflect changes in reward brain regions. Moreover, the changes in DNA methylation patterns were investigated at a single time point, thus, precluding the possibility of examining time-dependent effects of marijuana exposure on DNA methylation. Replication studies using specific cell types and brain tissues should help to further clarify the role of DNA methylation in CUD. Another issue that needs to be investigated in future replication studies include assessment of the role of marijuana on other epigenetic markers. This will entail examining changes in histone modifications and DNA methylation during the clinical course of repeated marijuana exposure. Indeed, the use of genome-wide methylation or chromatin immunoprecipitation studies should help to identify other genes that might be involved as either risk factors or consequences of cannabis exposure.

Overall, our results suggest a significant role of genes encoding proteins involved in the endocannabinoid system in the susceptibility for CU. Our study also identified epigenetic modifications affecting dopamine system function as risk factors and/or consequences of marijuana exposure. Confirmation studies that address the shortcomings detailed above are necessary to clarify to what extent changes in peripheral DNA methylation could serve as accessible sources of biological markers and/or targets for pharmacological interventions that might reduce or prevent the effects of marijuana in the brain. Finally, because education attainment was identified as a protective factor for CU, these observations suggest the building and expansion of early childhood educational programs and strategies to prevent CUD in adolescence and later in life.

## Electronic supplementary material

Supplementary material
