# Supplementary material for: Gene variants and educational attainment in cannabis use: mediating role of DNA methylation
Source: Transl Psychiatry. 2018 Jan 22;8:23. doi: 10.1038/s41398-017-0087-1 (PMC5802451; doi:10.1038/s41398-017-0087-1)
Supplement: Supplementary file 1 — Supplementary material [file 41398_2017_87_MOESM1_ESM.docx]

**SUPPLEMENTARY MATERIAL**

**Blood samples preparation**

1. *Biological samples collection*

The blood samples were previously collected and stored at -80°C for the project #12-DA-N472, NIDA, IRP [Health Outcomes by Neighborhood (HON) – Baltimore]. To achieve this research, approximately 5 mL aliquots of whole blood were provided for each subject.

1. *Genomic DNA extraction*

Blood samples were thawed at room temperature in a biosafety cabinet and immediately subjected to the DNA purification protocol from whole blood using the QIAamp DNA Blood Midi/Maxi Kit (Spin Protocol, QIAGEN). The blood samples were lysed with QIAGEN protease and Buffer AL. After lysis, the lysate is loaded onto the QIAamp spin column, DNA bound to the QIAamp membrane and impurities were washed away in two centrifugation steps. Finally, genomic DNA (gDNA) was eluted, reloading the elute twice to obtain maximum concentration.

1. *Aliquots preparation*

After DNA extraction, all the samples have been quantified with NanoDrop 2000c Spectrophotometer (Thermo Scientific) and divided into aliquots: 10 ng gDNA reserved for genetic analysis and 20 ug gDNA reserved for epigenetic analysis.

Genotyping procedure

Genotyping was performed in a volume of 5 μl containing 10 ng of gDNA: 2 μl volume of gDNA (5 ng/μl) for each sample were previously prepared in a 384-well plate and subsequently added 3-μL PCR Reaction mix (containing TaqMan Genotyping Master Mix, TaqMan genotyping assay mix, DNase/RNase-free water) in each well. Amplification was performed on a commercially available system (ViiA7, Life Technologies), starting with 30 sec at 60 °C, followed by 10 min at 95°C. Forty cycles of 15 s at 95 °C were than performed and 1 min at 60 °C. Genotypes were scored using the algorithm and software supplied by the manufacturer.

DNA methylation analysis procedure

20 ug of gDNA was sheared and subjected to denaturation by sonication to generate fragments between 300 bp and 600 bp. using the Diagenode Biorruptor (6 cycles, 30 s “ON”, 30 s “OFF”). A checking session has been run to decide the number of cycles to reach ~200–600 bp DNA fragments size. Six cycles session gave the most homogenously result. Systematic checking of the sheared DNA size on agarose gel to ensure equal sonication has been conducted after shearing session. The sheared samples were then processed for immunoprecipitation and qPCR.

**ANKK1, *Ankyrin Repeat and Kinase Domain Containing 1*gene, -0.25 kb TSS**

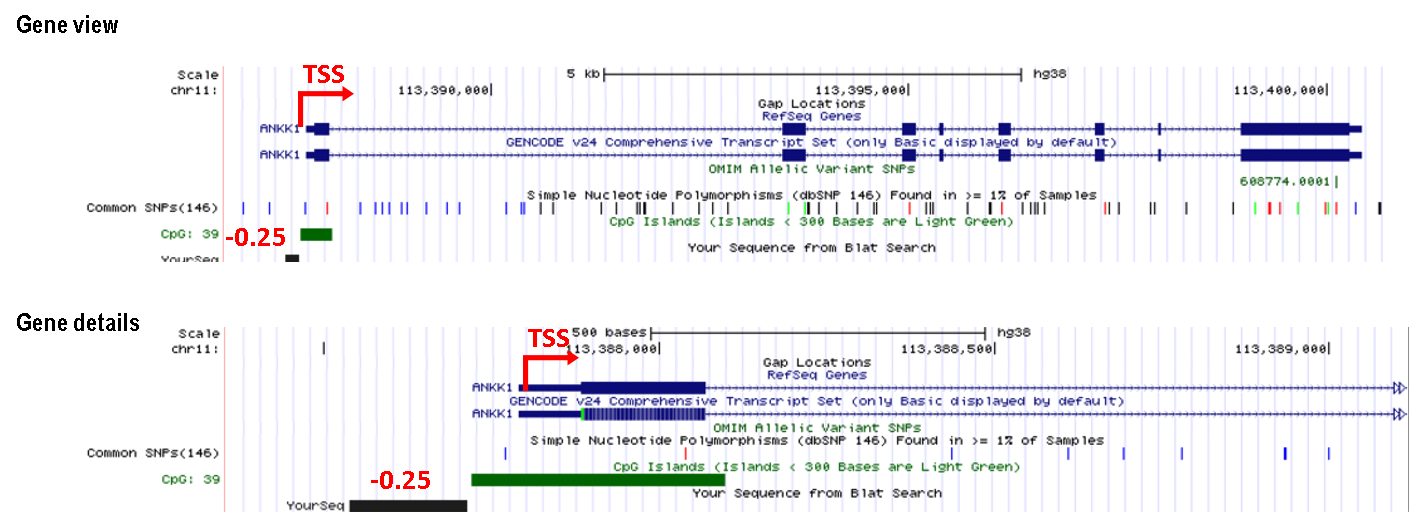


**Figure 1S.** Bar Plot for ANKK1 (-0.25 kb); Error bars: ±1 Standard error(s); unpaired t-test. Primers annealing region, -0.25 kb from TSS, ANKK1 gene (UCSC Genome Browser on Human Dec. 2013 (GRCh38/hg38) Assembly)

**DRD2, *Dopamine Receptor2*, -0.4 kb TSS**

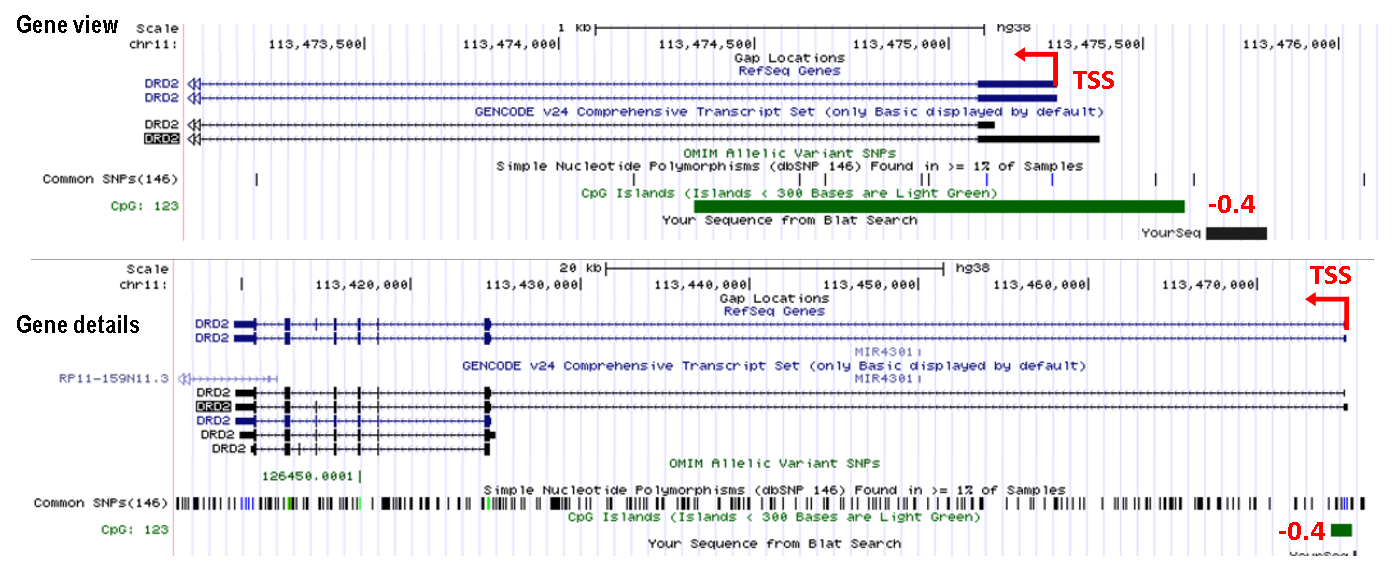


**Figure 2S.** Bar Plot for DRD2 -0.4 kb TSS; Error bars: ±1 Standard error(s); unpaired t-test. Primers annealing region, -0.4 kb from TSS, DRD2 gene (UCSC Genome Browser on Human Dec. 2013 (GRCh38/hg38) Assembly)

**DRD2, *Dopamine Receptor2*, +0.9 kb TSS on CpG**

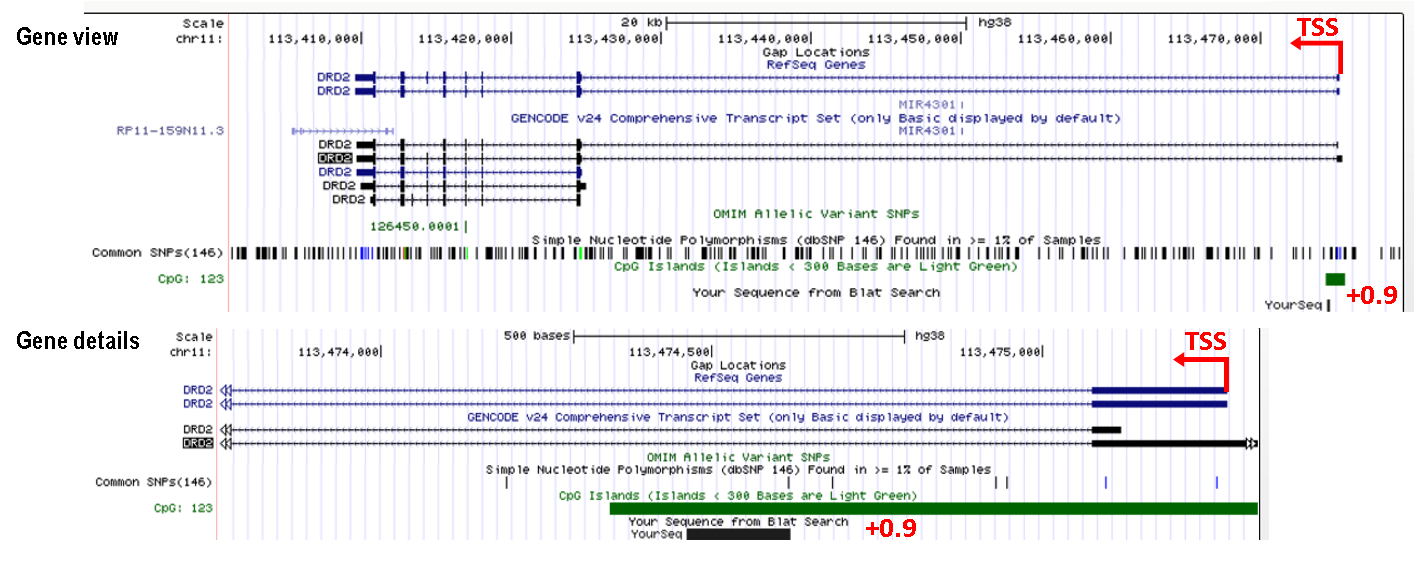


**Figure 3S.** Bar Plot for DRD2 (+0.9 kb TSS on CpG); Error bars: ±1 Standard error(s); unpaired t-test. Primers annealing region, +0.9 kb from TSS, DRD2 gene (UCSC Genome Browser on Human Dec. 2013 (GRCh38/hg38) Assembly)

**DRD2, *Dopamine Receptor2*, +66.7 kbTSS**

******

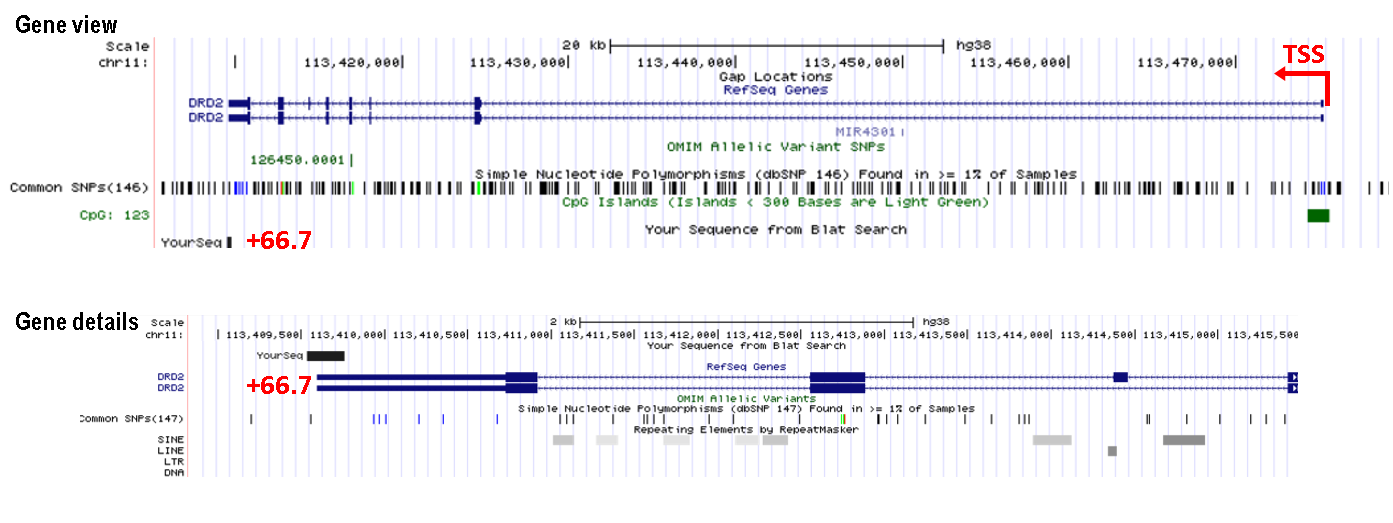


**Figure 4S.** Bar Plot for DRD2 (+66.7 kbTSS); Error bars: ±1 Standard error(s); unpaired t-test. Primers annealing region, +66.7 kb from TSS, DRD2 gene (UCSC Genome Browser on Human Dec. 2013 (GRCh38/hg38) Assembly).

**NCAM1, *Neural Cell Adhesion Molecule 1*, +0.4 kb TSS**

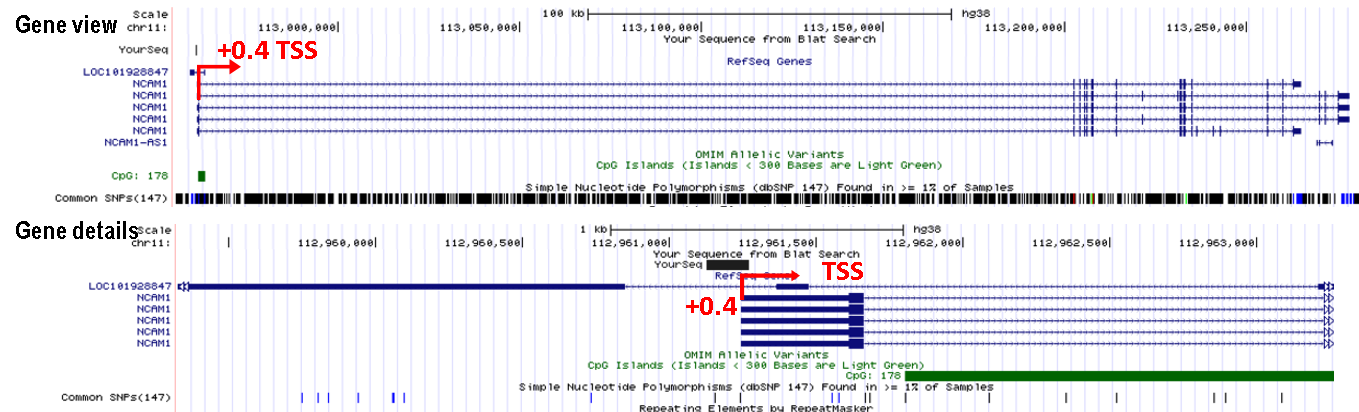


**Figure 5S.** Bar Plot for NCAM1 (+0.4 kbTSS); Error bars: ±1 Standard error(s); unpaired t-test. Primers annealing region, +0.4 kb from TSS, NCAM1 gene (UCSC Genome Browser on Human Dec. 2013 (GRCh38/hg38) Assembly)

**NCAM1, *Neural Cell Adhesion Molecule 1*, +3 kb TSS CpG**

*******

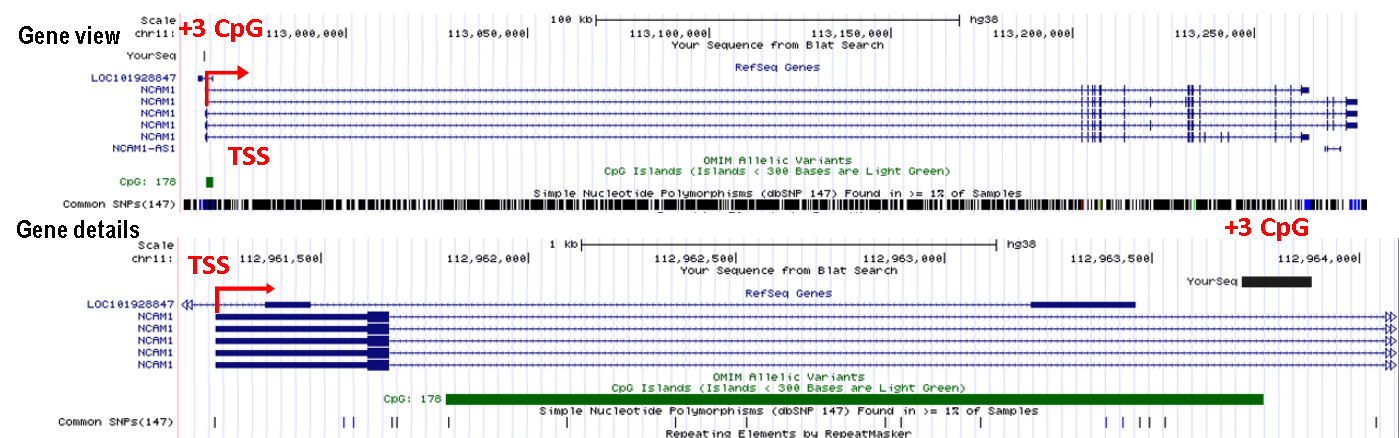


**Figure 6S.** Bar Plot for NCAM1 (+3 kb TSS CpG); Error bars: ±1 Standard error(s); unpaired t-test. Primers annealing region, +3 kb from TSS, NCAM1 gene (UCSC Genome Browser on Human Dec. 2013 (GRCh38/hg38) Assembly)

**CNR1, *Cannabinoid Receptor 1*, +22.31 kb TSS - rs1049353 SNP**

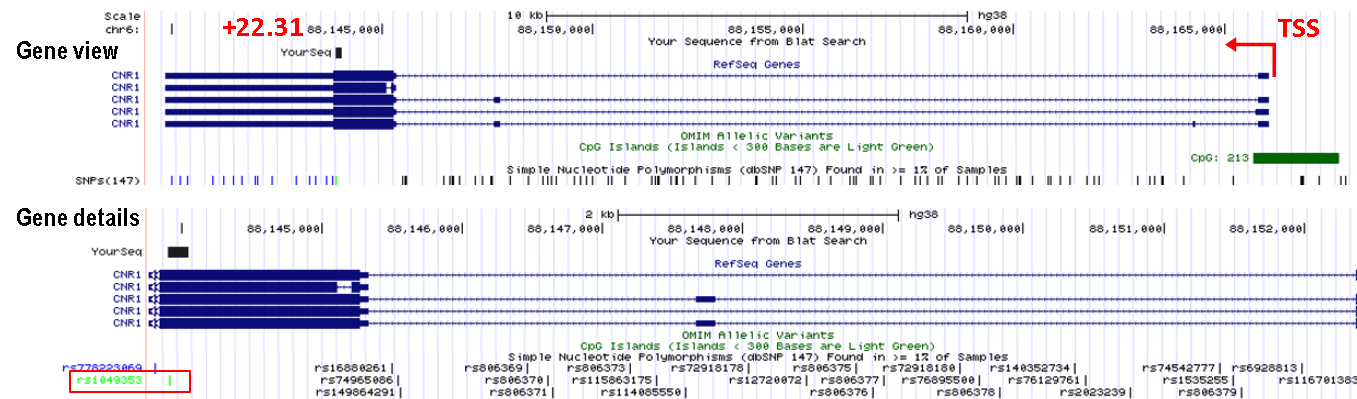


**Figure 7S.** Bar Plot for CNR1 (+22.31 kb TSS - rs1049353 SNP); Error bars: ±1 Standard error(s); unpaired t-test. Primers annealing region, +22.31 kb from TSS, CNR1 gene (UCSC Genome Browser on Human Dec. 2013 (GRCh38/hg38) Assembly)
